# Having a Lot of a Good Thing: Multiple Important Group Memberships as a Source of Self-Esteem

**DOI:** 10.1371/journal.pone.0124609

**Published:** 2015-05-27

**Authors:** Jolanda Jetten, Nyla R. Branscombe, S. Alexander Haslam, Catherine Haslam, Tegan Cruwys, Janelle M. Jones, Lijuan Cui, Genevieve Dingle, James Liu, Sean Murphy, Anh Thai, Zoe Walter, Airong Zhang

**Affiliations:** 1 University of Queensland, Brisbane, Australia; 2 University of Kansas, Lawrence, Kansas, United States of America; 3 Queen Mary, University of London, London, United Kingdom; 4 East China Normal University, Shanghai, China; 5 Victoria University of Wellington, Wellington, New Zealand; 6 Commonwealth Scientific and Industrial Research Organisation, Brisbane, Australia; The University of New South Wales, AUSTRALIA

## Abstract

Membership in important social groups can promote a positive identity. We propose and test an identity resource model in which personal self-esteem is boosted by membership in additional important social groups. Belonging to multiple important group memberships predicts personal self-esteem in children (Study 1a), older adults (Study 1b), and former residents of a homeless shelter (Study 1c). Study 2 shows that the effects of multiple important group memberships on personal self-esteem are not reducible to number of interpersonal ties. Studies 3a and 3b provide longitudinal evidence that multiple important group memberships predict personal self-esteem over time. Studies 4 and 5 show that collective self-esteem mediates this effect, suggesting that membership in multiple important groups boosts personal self-esteem because people take pride in, and derive meaning from, important group memberships. Discussion focuses on when and why important group memberships act as a social resource that fuels personal self-esteem.

## Introduction

A large body of work shows that people with more social group memberships have better psychological well-being, are healthier and live longer than those who belong to fewer social groups [1,2,3,4,5,6]. Much of this work has emphasized the contribution of social support in achieving these positive outcomes [7,8,9,10]. Yet while this research has generated important insights, the focus on social support has steered discussion away from the potential benefits that *mere belonging to important groups* can provide. That is, even though not all group memberships function as psychological resources for their members, many do. It is only those group memberships with whom people identify—that is, those that are *psychologically important* and internalized as part of social identity—that have the potential to boost self-esteem. This is because it is only when group memberships are a basis for defining the self that individuals can effectively utilize the psychological resources they provide. In the present paper, we will show that important group memberships serve as psychological resources [11,12,13] that have the capacity to boost self-esteem.

In examining these predictions, we draw on the social identity approach—comprised of social identity theory (SIT[14]) and self-categorization theory (SCT[15,16]). Previous research informed by this theoretical framework has tended to examine the ways in which group-level processes (e.g., group identification, group status and intergroup comparisons) affect personal self-esteem. The consequence is that interest in personal self-esteem among social identity researchers has waned in recent years and, as we outline below, it is no longer considered to be as central a construct as it was once.

### The Self-Esteem Concept in Social Identity Research

Historically, the construct of self-esteem was central to the social identity perspective. Indeed, the first assumption in the original statement of social identity theory was that “individuals strive to maintain or enhance their self-esteem; they strive for a positive self-concept” ([14], p. 40). During this early period it was argued that—because intergroup comparisons tend to be less favorable for lower- than for higher-status groups—members of lower-status groups derived less self-esteem from their group membership than did members of higher-status groups. As a result, members of lower-status groups engaged in a range of identity management strategies: either focusing on enhancing the status of their entire group (e.g., through social competition) or engaging in individual mobility with a view to gaining entry into the high-status group. Regardless of which identity-management strategy is used, social identity theory postulates that all strategies are aimed at achieving more favorable comparisons so as to arrive at a more positive social identity and, through this, enhanced self-esteem.

Even though this theorizing inspired much research, and led to a range of specific predictions [17], it became clear that the role of personal self-esteem in intergroup behavior was far from straightforward and quite complex [18]. Moreover, due to limited success in obtaining evidence that engagement in positive intergroup comparisons enhanced self-esteem, many have questioned its central role in motivating group behavior and have either abandoned it entirely [19] or downplayed its importance [20]. Although this development is understandable, we should bear in mind that past work was largely concerned with one specific question: whether or not engagement in identity management strategies (e.g., ingroup favoritism, emphasizing intergroup distinctiveness) enhanced personal self-esteem. However, that work did not examine what might be understood to be social identity theory’s most basic premise: that *mere identification* with a social group that is important to the self can boost personal self-esteem.

This is an important point, particularly given growing evidence that identification with social groups affects psychological well-being in positive ways [21,22,23,24]. For example, a large body of work has shown that among members of disadvantaged groups higher levels of group identification are associated with enhanced well-being [25,26,27,28,29]. Indeed, some of this work has provided evidence that group identification is related to personal self-esteem. For example, among a sample of African-Americans, Branscombe and colleagues [26] found that minority group identification was correlated with a number of well-being measures, including the Rosenberg Self-Esteem inventory [30]. More recently, Iyer, Jetten, Tsivrikos, Postmes, and Haslam [31] found that among a sample of British students the process of taking on a new student identity was associated with increases in personal self-esteem (measured by the Rosenberg Self-Esteem inventory) as well as greater life satisfaction and lower depression [32]. Given this growing body of work, it seems timely to assess the role of group membership in building personal self-esteem.

### Group Membership in Important Groups and Personal Self-Esteem

The social identity approach starts from the assumption that to understand individuals’ thoughts, beliefs and actions, we need to understand how they categorize themselves in relation to others. In particular, self-categorization theory argues that we can define ourselves in terms of our own unique individual traits and features (as ‘I”), or in terms of our social identity—our shared group membership with others (as ‘we’). When personal identity is salient, people focus on how the self, as an individual, is different from others, but when social identity is salient (e.g., our shared identity as women, Australians, or as employees of a particular organization) the focus shifts to the similarities with others who are part of one’s ingroup. Key here is the idea that what matters most is not whether one can legitimately lay claim to membership in a particular group but whether those group memberships are *psychologically internalized* and seen as relevant for understanding oneself and one’s place in the world (i.e., become self-categorizations, see [16]).

There are two important points that follow from this analysis. First, we argue that it is only when individuals identify with a group that the group becomes a psychological resource that individuals can utilize for the purpose of forming a positive social identity. This is because group identification and embeddedness not only provides a “ground to stand upon” ([33], p. 145) but also a sense of “existential security” [34]. It is on this basis that we predict that group identification is an important precondition for group membership to enhance a person’s sense of self-worth—their personal self-esteem [21].

Second, one way that we can test the hypothesis that social group memberships serve as a resource is by examining whether personal self-esteem (or well-being more generally) is enhanced as a function of the number of important group memberships to which individuals belong. That is, if group membership serves as a psychological resource, then multiple group memberships should enhance this resource such that every additional important group membership will increase, incrementally, one’s self-esteem. There is a growing body of work that supports ‘the more the merrier’ hypothesis [35,36,37,31] with recent research showing beneficial effects of multiple group membership when the psychological availability of multiple group memberships is varied through experimental manipulation ([38], Study 2). On this basis, we predict that personal self-esteem will be enhanced when people identify with multiple *and* important group memberships.

### Membership in Important Groups Affects Personal Self-Esteem through Collective Self-Esteem

To understand how personal self-esteem is affected by group membership, we need to consider the extent to which personal self-evaluations have a strong social and group-based origin [39,40]. Consistent with Cooley’s classic notion of the “looking glass” self [41], we argue that self-evaluations are largely reflections of the views of important others—in particular, those in the groups to which we belong. Especially relevant for our purposes is evidence showing that the self-esteem we derive from membership in social groups—our collective self-esteem [42,43,44]—serves as an important basis for personal self-evaluations and personal self-esteem. In support of this case, research shows that collective self-esteem is associated with enhanced personal well-being [45] and better health [46].

When individuals identify with important groups, those group memberships (and the social identities they support) provide a common perspective on social reality and a lens through which to perceive and understand the world [21,47]. This is because individuals come to understand themselves not just as individuals (as ‘I’ and ‘me’), but as part of a larger collective (as “we’[14]). Put differently, when individuals identify with groups, they derive collective self-esteem from those groups because identification enhances the perception of a shared outlook on life and furnishes people with a sense of purpose and belonging—which should in turn have positive consequences for their personal self-esteem. In this way, collective self-esteem can be seen as a marker of people having drawn on the resources that group memberships (and associated social identities) offer.

Consistent with this reasoning, there is evidence that social identification enhances collective self-esteem [48,49,32] and that countering a group threat elevates collective self-esteem [50,51]. In the present research, we therefore test the hypothesis that collective self-esteem mediates the relationship between social identification with multiple important groups and personal self-esteem.

### Interpersonal Ties versus Multiple Important Group Memberships

It is important to acknowledge that a large body of research has pointed to the beneficial effects for well-being that result from having a larger number of interpersonal ties [52,53] and a greater quantity of social interactions [54]. However, this research typically does not examine separately the effects of the number of group memberships and the number of interpersonal relationships (e.g., number of friends) on indicators of well-being. For example, Christakis and Fowler [52,53] count the number of contacts as person-to-person ties, taking into account whether these contacts are one-way or two-way. Others ask participants to list the number of roles they have and relate those to the amount of contact individuals have [35] or ask participants to think about the number of people they typically engage in within these roles over a period of two weeks [55].

We believe that it is important to distinguish the effects of the number of interpersonal ties (either as part of role interactions or not) and the number of group memberships in order to assess whether group memberships are a stronger predictor of personal self-esteem than the number of interpersonal ties that a person reports. This is an important distinction because it is only through group identification that the group and other group members become *part of the self*, and are encompassed within an individual’s social identity [15]. Given that identification with the group unlocks unique psychological resources such as a sense of belonging, purpose, meaning, and support, group memberships are better suited than interpersonal ties to act as a social resource [21,56,57].

### Overview of Research

These arguments inform a set of hypotheses that the present research was designed to test. First, we hypothesise that the relationship between the number of group memberships and personal self-esteem will be predicted by a ‘more the merrier’ effect—such that the more group memberships a person has, the greater their self esteem. Importantly, though, we argue that individuals will derive personal self-esteem from a group membership—and will be able to benefit from the purpose, meaning and sense of belonging that group membership provides—only to the extent that they identify with that social group or category (H1). Second, differentiating our social resource model from research examining the benefits of interpersonal ties, we predict that the relationship between multiple and important group memberships and personal self-esteem will be stronger than the relationship between number of interpersonal ties and personal self-esteem (H2). Finally, we propose that collective self-esteem will mediate the relationship between important group memberships and personal self-esteem. This is because we take collective self-esteem as a sign that people are benefitting from the psychological resources that group memberships provide and these resources are a basis for enhanced personal self esteem (H3). We test these hypotheses in eight studies across a wide range of groups and cultural contexts.

In our examination of the relationship between group membership and personal self-esteem, we recognize how important it is to be sensitive to the context in which self-esteem is measured. Self-esteem may be less valued in some cultures [58] and populations [59]. Accordingly, we assess self-esteem by tapping self-esteem components that are most relevant in a particular cultural or group setting. In line with Tafarodi and Swann [60], we measure personal self-esteem as either *self-liking* (as measured by the Rosenberg Self-Esteem inventory, and the one-item self-esteem measure [61]) or as *self-competence*. While we measure self-esteem in most studies as self-liking, there are two exceptions to this. First, in line with Markus and Kitayama’s [58] reasoning that self-esteem is a concept that is more relevant in Western than in Eastern contexts, we measure self-esteem as *self-satisfaction* among older adults in China (Study 1b) and as *self-competence* among university students in China (Study 3a). Second, we chose to measure self-esteem as *self-competence* in vulnerable samples (e.g., as measured by personal identity strength in the study among residents of a homeless shelter [62]), because this seemed more appropriate given the high levels of mental illness in this group.

In the first three studies, we examine the relationship between multiple group memberships and personal self-esteem across the age range (children in Study 1a and older adults in Study 1b) and populations whose self-esteem might be compromised due to age (older adults in Study 1b) and disadvantaged living circumstances (children in Study 1a and residents of a homeless shelter in Study 1c). In the first three studies, to better understand the validity of our main predictor multiple important group memberships, we measured multiple important identities in two ways and examined the relationship between each measure and personal self-esteem. Specifically, we employed a measure that involved participants indicating their agreement with items assessing whether they belonged to many important groups (Exeter Identity Transition Scale; EXITS [37]) as well as a measure on which participants were asked to list the number of groups with which they identified strongly. Previous research has shown that both indices correlate in similar ways with well-being measures that tap life-satisfaction [37] and in Study 1a we investigated whether this was also true for measures relating to personal self-esteem.

A second aim of the first three studies was to examine the correlates of multiple group memberships and to assess the extent to which they affected the relationship between multiple important group memberships and personal self-esteem. In particular, recent research has found that people with high SES (assessed in terms of social class or level of education) have more social capital—that is, they have larger social networks [31,63,6]. Here, we examine the extent to which social class and education affect the number of important group memberships to which participants belong and control for their effects. Finally, in these studies, we measure personal self-esteem in different ways: as self-liking in the sample of children (Study 1a) and as self-satisfaction (Study 1b) and self-competence (Study 1c) because this was perceived to be more appropriate given each specific context.

In Study 2, we test our prediction that membership in social groups is a stronger predictor of personal self-esteem than social network indicators such as the number of interpersonal ties (H2). Among boys in a large school we were able to conduct a complete social network analysis [64] allowing us to compare the contribution of the effects of multiple group memberships on personal self-esteem with the relationship between the number of interpersonal ties and personal self-esteem.

To provide evidence for directionality, Studies 3a and 3b examine the relationship between multiple group memberships and personal self-esteem over time. Specifically, Study 3a examines over two measurement times whether multiple important group memberships determine personal self-esteem, or vice versa. Study 3b examines over three time points whether change over time in multiple important group memberships predicts self-esteem at T3.

Studies 4 and 5 examine the prediction that collective self-esteem mediates the relationship between multiple important group memberships and personal self-esteem (H3). The methodology in the final two studies differs from that of the previous studies. Here, in relation to a number of groups, participants are asked to indicate the extent to which they identify with each before completing collective and personal self-esteem measures. This procedure allows us to test our hypotheses while keeping the type of the groups that participants considered constant.

## Study 1a: Multiple Group Membership and Self-esteem in Children

Our first study examined the relationship between multiple group memberships and personal self-esteem among children attending a primary school in Britain. The school was located in a relatively disadvantaged working class neighbourhood experiencing high levels of unemployment. We first examined the convergent validity of our measure of multiple important group memberships by exploring whether scores on items tapping the extent to which participants felt they belonged to many important groups (Exeter Identity Transition Scale; EXITS, [37]) corresponded with the number of group memberships that they listed as important to them. Having done this, we then examined the relationships between these two measures of multiple group memberships and personal self-esteem.

Building on previous research that has shown that Socio-Economic Status (SES) is an important predictor of multiple group memberships in adults [63], we also examined the relationship between multiple group memberships and personal self-esteem while controlling for SES as rated by the children’s parents.

### Method

#### Participants

Participants were 29 children and their parents who were recruited from three primary schools in Cornwall, UK. There were 11 male and 18 female children with an average age of *M *= 10.88 years (*SD* = 0.68). Associated questionnaires were also completed by each child’s parent (24 mothers and 5 fathers) with an average age of *M *= 40.82 years (*SD* = 4.75).

#### Ethics Statement

This study obtained ethical clearance from the ethics committee at the School of Psychology, University of Exeter (UK). After parents or guardians provided their written consent, participating children completed their questionnaires in the classroom during school hours.

#### Procedure and Measures

Multiple group membership was assessed with three items from the Exeter Identity Transition Scale (EXITS; [37]). These included: “I am a member of lots of different groups”, “I am active in lots of different groups”, and “I have friends in lots of different groups” (1 = *Do not agree*, 7 = *Agree completely*; *α* = .84).

To assess the convergent validity of the multiple group membership measure, participants were also asked to list up to six different groups to which they belonged. Participants were then asked to look back over the groups they had listed and to rate the importance of each group listed (1 = *Not important*, 7 = *Very important*).

*Personal Self-Esteem* (PSE) was assessed using an abbreviated and simplified seven-item scale adapted from Rosenberg [30] that included both positive (i.e., “I am able to do things as well as most people”) and negative items (i.e., “I feel that I am a failure”; 1 = *Strongly disagree*, 7 = *Strongly agree*). Negative items were reverse-scored so that high scores indicated higher levels of PSE (*α* = .85).

Participating children’s responses were complemented by data provided by their parents that assessed SES. Specifically, parents were asked to indicate how they perceived their own family’s class on a 9-point scale (1 to 3 = *working class*, 4 to 6 = *middle class* and 7 to 9 = *upper class*). This measure was taken from Jetten and colleagues [65] who developed it for use in a similar cultural context.

### Results and Discussion

Bivariate correlations between variables are displayed in Table 1. There was a positive correlation between the EXITS measure of multiple group memberships and PSE, *r* = .48, *p* = .008, indicating that the more participants agreed that they belonged to multiple groups, the higher their reported PSE. Moreover, multiple group memberships were positively correlated with SES as rated by the participants’ parents (*r* = .42, *p* = .028). The magnitude of the relationship between the EXITS measure and PSE increased when controlling for SES (*r*_*p*_ = .61, *p* < .001).

**Table 1:** Study 1a: Descriptive statistics and bivariate correlations for British children.

	*M (SD)*	1	2	3	4	5
1. Multiple group memberships	5.37 (1.69)	1	.61**	.76**	.79***	
2. Personal self-esteem	5.41 (1.15)	.48*	1	.41*	.62**	
3. Number of groups listed	4.21 (1.68)	.79**	.36	1	.87***	
4. Number of groups X importance	23.69 (10.59)	.84***	.49**	.92***	1	
5. Socio-Economic Status	4.89 (.93)	.42*	.43*	.35	.33	1

*Note*.

*p < .05,

**p < .01,

***p < .001.

Bivariate correlations are presented below the diagonal. Partial correlations (controlling for Socio-Economic Status as rated by the participants’ parents) are reported above the diagonal.

Further analyses focused on the relationship between scores on the multiple group membership scale and the groups that were listed by the participants (both as the number that they listed and by creating a new score by multiplying the number listed by the rated importance of the listed groups). A number of findings are noteworthy. First, the group memberships reported by the children were overwhelmingly leisure-based (i.e., sporting and musical groups, 91%). There was limited involvement amongst children in educational or ideology-based groups such as churches (4% and 3%, respectively).

Second, there was a strong correlation between participants’ multiple group membership EXITS scores and the number of groups that they listed, *r* = .79, *p* < .001 (and this correlation was largely unaffected by the rated importance of these groups, *r*_*p*_ = .76, *p* < .001), and the number of groups participants listed multiplied by their importance, *r* = .84, *p* < .001. This suggests that children were thinking of groups that were important to them when they were completing the EXITS multiple group membership scale. More generally, this provides reassurance about the convergent validity of the scale—in so far as the multiple group memberships scale appears to reflect the number of important social groups that participants belong to. Third, there was a significant relationship between number of important groups that participants listed and PSE, *r* = .49, *p* = .007, a relationship that became stronger when we controlled for SES, *r*_*p*_ = .62, *p* = .001.

Results of Study 1a provide support for H1 and indicate that belonging to a greater number of important groups is associated with feeling better about one’s self. Interestingly too, and in line with previous work [31,63], SES, as rated by the participants’ parents, was an important predictor of the number of groups to which children said they belonged [63]. However, when we controlled for SES, the relationship between multiple important group membership and PSE remained significant (and in fact became stronger). This suggests that SES does not account for this relationship. Consistent with our assumption, this study also confirmed that our multiple group membership measure (i.e., the EXITS) was essentially a proxy for the number of group memberships that respondents identified with strongly. Specifically, the correlation between the number of group memberships that participants listed (multiplied by their importance) and their scores on the multiple group membership scale was high, and both correlated similarly with PSE.

## Study 1b: Multiple Group Membership and Self-Esteem in Older Adults

In our second study, we examined the relationship between multiple group membership and PSE among retirees in China. Older adults have been found to be at risk of isolation after retirement, in particular when social participation is increasingly limited by physiological and psychological decline [66]. Accordingly, we examined the relationship between multiple group membership and personal self-esteem while controlling for SES (here measured as level of education, [67]).

### Method

#### Participants

Participants were 131 retired older adults primarily living in Shanghai (*N* = 100), with the remainder from 4 other Chinese cities: Chengdu, Hebei, Jilin, or Tianjin. Seven participants had missing data on many key variables and were therefore omitted from the analyses, leaving 124 participants in the sample (65 males, 54 females, 5 missing) with an average age of *M *= 67.81 years (*SD* = 9.46, ranging from 52 to 89, 4 missing values). Most participants lived with their spouse (*N* = 113, 91.1%, 3 missing values) and they reported having an average of two children (*M *= 1.86, *SD* = 1.00, ranging from 0 to 5, with 8 missing values). A large proportion of the participants (*N* = 53, 42.7%, 4 missing values) indicated that they lived with their children.

#### Ethics Statement

This study obtained ethical clearance from the research committee at the East China Normal University. Because many participants had limited literacy skills and/or poor eye-sight, verbal informed consent was obtained, recorded and written down by the researcher. Questions in the survey were read out to participants. This procedure was approved by the research committee at the East China Normal University.

#### Procedure and Measures

All measured items were developed in English and then translated into Chinese. The Chinese version was then back-translated into English by a professional translator. Any differences in the translations were discussed until agreement was reached. Data were collected after special seminars for the aged at retirement villages in Shanghai. Additional data were collected by students in other cities.

Because Study 1a confirmed that our multiple group membership measure tapped the number of group memberships that are important to participants, multiple group membership was measured with the same three items (EXITS; *α* = .76). In line with the suggestion by Markus and Kitayama [58] that personal self-esteem is a concept that is more relevant in Western than in Eastern contexts and their recommendation that self-satisfaction is more culturally appropriate to assess in the East, we assessed personal self-esteem with an item adapted from the Quality of Life questionnaire [68]. This item asked participants to complete the statement “when I think about myself as a whole, I feel I am…” with one of the following response options: “*poor”*, *“fair”*, *“good*”, or “*excellent*”. While it might be argued that personal self-esteem is best measured with multiple items, this one item measure is not only valid but produces similar findings to multi-item scales [61].

Socio-economic status is often conceptualized as level of education [69] and this appeared to be the most appropriate index of SES in this cultural context. One item was included asking participants to indicate their highest level of education on a scale ranging from (1) “*Basic forms of education*” to (6) “*A university degree*.”

### Results and Discussion

Preliminary analyses indicated that gender, number of children, and whether participants were living with a spouse or children did not affect the relationships between key variables. Accordingly, these measures were omitted from further analyses. Bivariate correlations between key variables are displayed in Table 2.

**Table 2:** Study 1b: Descriptive statistics and bivariate correlations for older adults in China.

	*M (SD)*	1	2	3
1. Multiple group memberships	3.28 (.71)	1	.29**	
2. Personal self-esteem	2.98 (.60)	.23*	1	
3. Socio-Economic Status	4.07 (1.09)	.09	.41***	1

*Note*.

*p < .05,

**p < .01,

***p < .001.

Bivariate correlations are presented below the diagonal. The partial correlation between multiple group membership and personal self-esteem (controlling for Socio-Economic Status) is reported above the diagonal. Correlations are based on sample sizes varying from *N* = 109 to *N* = 124.

Consistent with H1, multiple group memberships and PSE were positively correlated, *r* = .23, *p* = .015, indicating that the more participants reported belonging to multiple groups, the higher their reported PSE. In contrast to Study 1a, multiple group membership did not correlate with SES (*r* = .09, *p* = .356). This may reflect the fact that most participants were well educated; therefore the limited variance in the sample may explain the absence of a correlation. However, and in line with both Study 1a and previous research [63], SES was a strong predictor of PSE—with higher levels of SES (i.e., education) associated with higher PSE (*r* = .41, *p* < .001). Finally, and as in Study 1a, the magnitude of the relationship between membership in multiple group membership and PSE increased slightly when controlling for SES (*r*_*p*_ = .29, *p* = .003).

## Study 1c: Multiple Group Membership and Self-Esteem among Homeless People

To determine whether the relationship between multiple group memberships and personal self-esteem would also be observed among a sample that is extremely disadvantaged, we tested H1 among residents in crisis accommodation for the homeless at three time points. First, when they were in the crisis centre (T1), then three months (T2) and a year (T3) after they had left. At T1, we asked participants to consider the number of groups they belonged to since entering the crisis centre, and then at T2 to indicate the extent to which they had acquired many new group memberships since T1. We then examined relationships between these different assessments of multiple group membership and personal self-esteem (indexed as self-satisfaction and personal identity strength at T3). In consultation with our industry partner, The Salvation Army (who allowed us to conduct research in their homeless shelters), we decided not to include self-esteem measures that tap self-liking because this was seen as inappropriate in light of the sample’s vulnerability. In this context, it was deemed to be more suitable to tap self-satisfaction and personal identity strength.

In this study, we also asked participants at all three time periods to list the significant other people in their lives. Because we predicted that the beneficial effects of social connectedness for self-esteem are only observed when participants identify with groups (such that they are a basis for self-categorization), we expected that it would be multiple important group identities and not the number of individuals in a person’s life that would predict personal self-esteem.

### Method

#### Participants

Participants were recruited as part of a larger project from six Salvation Army homeless accommodation shelters across southeast Queensland in Australia. Each shelter provides accommodation for people who present as homeless or at risk of homelessness. The maximum length of stay in the crisis shelter is 12 weeks, although this can be extended at the discretion of the shelter workers. To recruit participants, the researchers described the study to residents during group meetings, and invited all residents to participate. Some participants were also recruited via word of mouth from other participants or staff. Individual interviews were scheduled for residents who indicated they were interested in taking part in the study.

A total of 119 participants completed the questionnaire at T1, including 56 men and 63 women, with an average age of 35.4 years (range 19–59; *SD* = 9.3). At T1, the average time participants had been in the homelessness shelter was 7.5 weeks. T2 data (*n* = 76) was collected from those who could be located 3 months later (or 2 weeks after leaving the shelter) and T3 data (*n* = 44) was collected 12 months from T1. Among those taking part at T2, 54% were in stable accommodation, while 60% were in stable accommodation at T3. At T1, 18.5% of participants had some form of paid employment and 90% were in receipt of government benefit, compared with 26% and 80.5% at T2, and 16% and 86.5% at T3. We found no evidence for systematic attrition on key measures and results are reported for those 44 participants (11 men, 33 women, average age of *M *= 36.41 years, *SD* = 9.01) who completed the measures at all three time-points (MCAR test: χ(29) = 22.44, *p* = .802).

#### Ethics Statement

This study obtained ethical clearance from the Behavioural and Social Sciences Ethical Review Committee (BSSERC) at the University of Queensland. Before completing the questionnaire, participants were informed about the aims of the study, and informed consent (both verbal and written) was obtained.

### Procedure and Measures

If low literacy was an issue, questions were read aloud by the researchers to which participants responded verbally. At T2 and T3, researchers met with participants at a location of their choosing (e.g., the participant’s home, a coffee shop). At each time-point, participants were compensated AU$20 for their time.

#### Multiple Group Memberships Since Transition to the Shelter (T1)

Participants were asked to indicate their agreement with the items: “Since coming to [name of accommodation shelter], I am a member of lots of different social groups” and “Since coming to [name of accommodation shelter], I have friends who are in lots of different groups” (*r* = .66, *p* < .001). Given the need to keep the survey as short as possible, abbreviated scales were used; only two items were included in this study whereas three items were used in Studies 1a and 1b (see Jetten et al., [70] for a similar procedure).

#### Multiple Group Memberships Since Transition out of Shelter (T2)

Participants were asked to indicate their agreement with the items: “After living at [name of accommodation shelter], I am a member of lots of different social groups” and “After living at [name of accommodation shelter], I have friends who are in lots of different groups (*r* = .78, *p* < .001).

#### Multiple Group Memberships at T3

Participants were asked to respond to the single item: “I am a member of lots of different groups” (multiple groups T3). Answers on these multiple group membership measures were provided on scales ranging from (1) “*Do not agree at all*” to (7) “*Agree completely”*.

#### Number of Interpersonal Ties

To assess number of interpersonal ties, at each measurement time, participants were asked to write the names of the people that were important in their lives and the number of people that were listed was counted (network size T1, T2 and T3).

#### Personal Self-Esteem

Personal Self-Esteem was assessed in two ways. First, self-satisfaction was measured with a single item from the Warwick-Edinburgh Mental Well-being scale (Item 8; [71]) asking participants to reflect on the last two weeks and indicate to what extent they agreed with the statement “I have been feeling good about myself”. Second, personal identity strength was assessed using three items adapted from Baray and colleagues ([62], 1 = *Strongly disagree*, 7 = *Strongly agree*). These items were: “I feel that I have a clear goal in my life”, “I know what future I want to pursue”, and “I have a clear idea about what is important and unimportant”. The items were averaged, with higher scores indicating higher personal identity strength (α = .91).

### Results and Discussion

Participants reported belonging to a moderate number of groups since entering the shelter at T1 (*M* = 3.43, *SD* = 1.77) and this did not change substantially after leaving the shelter (T2: *M* = 3.17, *SD* = 1.74; T3: *M* = 3.47, *SD* = 1.80; see Table 3 for descriptive statistics and correlations). Participants reported moderate levels of personal self-satisfaction (*M* = 3.37, *SD* = 2.00, possible range from 1 to 5) and personal identity strength (*M* = 5.56, *SD* = 1.33, possible range from 1 to 7).

**Table 3:** Study 1c: Descriptive statistics and bivariate correlations for homeless adults in Australia.

	*M (SD)*	SS	PIS
Multiple group memberships:			
since transition to shelter (T1)	3.43 (1.77)	.10	.05
since transition out of shelter (T2)	3.17 (1.74)	.35*	.37*
at T3	3.47 (1.80)	.31*	.39**
Number of interpersonal ties (T1)	4.95 (1.96)	.07	.06
Number of interpersonal ties (T2)	5.24 (2.09)	.21	.10
Number of interpersonal ties (T3)	5.37 (2.12)	.10	.20
Self-satisfaction (T3)	3.37 (.82)	1	.48**
Personal identity strength (T3)	5.56 (1.33)	.48**	1

*Note*.

*p < .05,

**p < .01.

SS = Self-satisfaction and PIS = Personal Identity Strength. Correlations are based on sample sizes varying from *N* = 38 to 44.

We examined the relationship between multiple group memberships and number of interpersonal ties with self-satisfaction and personal identity strength in two ways. First, using multi-level modeling (STATA package 12.1), we ran two models comparing the power of multiple group memberships at all 3 time-points and number of interpersonal ties at all 3 time-points to predict self-satisfaction (Model 1) and personal identity strength (Model 2). Consistent with the correlations, multiple group membership but not number of interpersonal ties predicted self-satisfaction at T3 (Model 1, B = .11, SE = .04, Z = 3.01, *p* = .003 and B = .03, SE = .03, Z = 1.12, *p* = .262, respectively). In a similar vein, multiple group membership but not number of interpersonal ties predicted personal identity strength at T3 (Model 2, B = .19, SE = .06, Z = 3.19, *p* < .001 and B = .03, SE = .04, Z = .57, *p* = .568, respectively).

Second, we examined how change over time in multiple group membership and number of interpersonal ties predicted self-satisfaction and personal identity strength. Specifically, we calculated two gain scores by subtracting T1 from T2 multiple group membership and number of interpersonal ties and correlated this with T3 self-satisfaction and T3 personal identity strength. Analysis revealed that gains in multiple group membership was marginally significantly positively related to T3 self-satisfaction (*r* = .303, *p* = .064) but that gains in number of interpersonal ties was not (*r* = .083, *p* = .622). Furthermore, gains in multiple group membership were positively related to T3 personal identity strength (*r* = .363, *p* = .025) but gains in number of interpersonal ties were not (*r* = .071, *p* = .672).

In sum, and consistent with H1, multiple group memberships *did* predict self-satisfaction and personal identity strength 12 months after leaving the shelter (T3). What is more, this study provides evidence consistent with H2 that while multiple group membership predicted self-esteem measures at T3, number of interpersonal ties did not. There was also evidence that gains in multiple group membership over time predicted self-satisfaction (albeit only marginally significantly) and personal identity strength at T3. Gains in the number of interpersonal ties did not predict personal self-esteem measures at T3.

### Summary of Studies 1a, 1b and 1c

Consistent with H1, in three different countries (UK, China, Australia) across three different samples, each facing different challenges (i.e., social disadvantage, aging, homelessness), we found a significant relationship between belonging to multiple groups and personal self-esteem. This was also true (and in fact was typically more true) when controlling for potentially relevant covariates (social class and education). Importantly too, consistent with H2, personal self-esteem was not associated with number of important interpersonal ties.

## Study 2: Number of Important Groups versus Number of Friends

Supporting H2, Study 1c provided preliminary evidence that personal self-esteem and personal identity strength are predicted by the number of important group memberships that a person has (in line with H1) and not by the number of important interpersonal ties. This hypothesis was explored further in Study 2 among a sample of boys at a private school in a large city in Australia. Here we again tested our prediction that it would not be the number of friends at school (i.e., number of interpersonal ties), but the number of groups that one is highly identified with, that best predicts personal self-esteem (H2). To provide a fair test of our predictions, we focused only on group memberships within the school, and not on those outside the school, and compared this with a social network analysis indexing number, structure, and quality of interpersonal ties within the school.

### Method

#### Participants and Procedure

Participants were 827 boys who attended a large private school in Australia. For a small number of participants it was not possible to calculate network scores because names could not be matched up, so only those participants for whom these data could be obtained were included—reducing the sample to 813 (year 8, *n* = 174, year 9, *n* = 177, year 10, *n* = 214, year 11, *n* = 161 and year 12, *n* = 87). Age ranged from 12 to 16 years. Of these 813 participants, 126 (16%) were boarding at the school. Participants completed their questionnaires during school hours.

#### Ethics Statement

This study obtained ethical clearance from the Behavioural and Social Sciences Ethical Review Committee (BSSERC) at the University of Queensland. Participants ticked a box before they started completing the surveys indicating their (written) informed consent.

### Measures

Multiple group membership was assessed using the same EXITS items as Study 1a (α = .88). Participants were also asked to name up to six different groups to which they belonged and to rate the importance of each of these groups. We coded whether groups were related to the school (e.g., athletics team, chess club, etc) and used only these to calculate group number and importance. The number of groups listed was used as a separate measure, but we also multiplied this measure by the importance of these groups to create an index of the number of important groups in the school.

*Interpersonal ties* were calculated based on an open name generator in which students were asked to nominate their “closest friends in [name of school].” Students filled out online questionnaires in which they were first asked to name up to five friends. If five names were given, students were provided with the option to name up to an additional five friends. Year 10 students completed a paper version of the same questionnaire that had 10 blank spaces for friend’s names. The names students gave were then matched by coders to a school roster and converted to student ID numbers (only friend nominations that could be matched to other students in the school were counted). Three local network measures of friendship were then calculated; *In-degree*, the number of students who named a participant as a friend, *Out-degree*, the number of friends a student named, and *Degree*, the sum of in-degree and out-degree [72].

**Fig 1:**
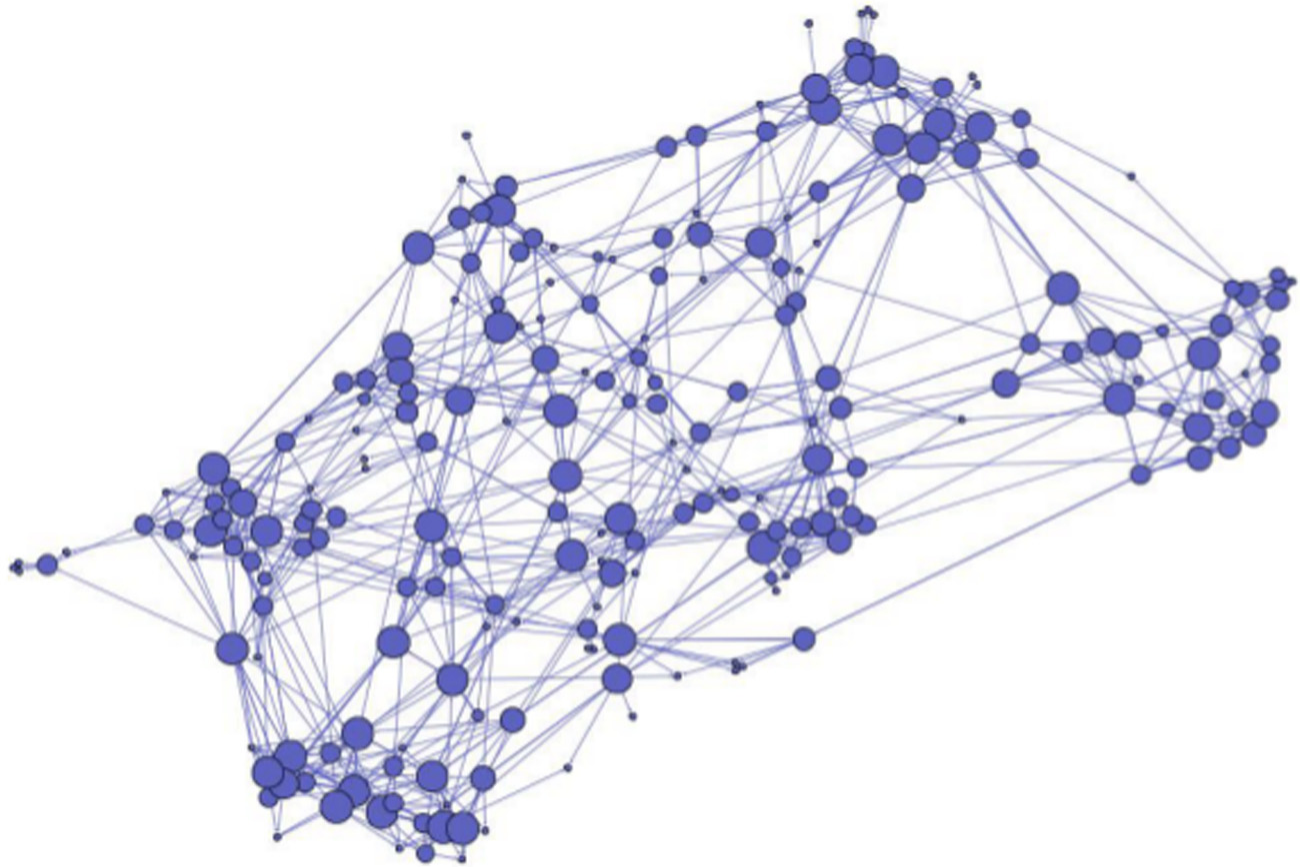
Study 2: Network representing *out-degree* (i.e., the number of friends a participant claimed) for Year 11 participants at a boys’ school in Australia. *Note*. Every participant is represented as a node and the size of the node represents the number of friends that were listed by participants. Connections between nodes indicate interpersonal ties. *N* = 161.

A complete network [64] was then formed for all responders within each grade, with each student forming a node and friendship ties forming edges between nodes. To create this network, we used undirected edges [73], creating a link between two students if either one named the other as a friend. As an illustration, the complete social network for the Year 11 participants is presented in Fig 1, with each node sized relative to the out-degree of the student it represents. Cross-grade friendships represented a very small percentage of friendships named and were not included when calculating the networks (unless, as stated in Footnote 7, individuals outside the year level were named by more than one individual, in which case those individuals, but not the rest of their year level, were included for the purposes of calculating the network measures before being removed). Each grade level network was calculated separately.

From this network, we calculated two global network measures of friendship. The first, *number of triangles*, was a measure of how many triadic closures were formed by a student’s friendship ties (i.e. how many pairs of a student’s friends were themselves friends). Triadic closures have long been theorized to represent especially strong friendships [74], so this measure captures not just the number but also the quality of each student’s friendships. A second measure calculated *closeness centrality*, the inverse of the average number of steps from a participant to each other participant in the network [72,64]. Closeness is a measure of the extent to which a participant is well connected within the network, measuring the ease with which he or she could reach any other through friendship ties. Overall, then, we calculated five separate indices of a person’s interpersonal network that could be assessed as potential predictors of personal-self-esteem.

*Personal Self-Esteem* (PSE) was assessed with one item [61]: “I have high self-esteem” (scale ranging from 1, *Strongly disagree* to 4, *Strongly agree*).

### Results and Discussion

Because initial examination indicated that more friends were named by Year 10 students who completed the hardcopy survey, network measures for these students were mean-centred separately from the other students before analyses. There were no interactions between Year 10 membership and any network measure in predicting PSE, and so all years were subsequently examined together. Links to unmatched names that were provided by more than one participant, as well as links to matched names of students who did not complete the friendship questionnaire, were included in the network for completeness in calculating network measures, and then were removed.

We first examined the bivariate correlations between PSE and both indicators of multiple important group memberships (EXITS measure, number of groups listed, number of important groups) and the five network indices of interpersonal ties (see Table 4). Consistent with H1, examination of bivariate correlations revealed that the EXITS measure of multiple group memberships, number of groups listed, and number of important groups listed all correlated positively and significantly with PSE. However, even though out-degree, degree, and number of triangles were positively correlated with PSE, the correlations of the two other network measures (in-degree and closeness centrality) with PSE were not significant.

**Table 4:** Study 2: Descriptive statistics and bivariate correlations, for children/adolescents in a boys’ school in Australia.

	*M (SD)*	Personal self-esteem
Personal self-esteem	3.11 (.70)	1
EXITS Multiple group		
memberships	5.07 (1.42)	.29***
Number of groups listed	2.34 (1.48)	.13***
Number of important groups	14.31 (7.59)	.17***
In-degree	5.78 (3.85)	.05
Out-degree	6.94 (2.64)	.12**
Degree	12.72 (5.48)	.10**
Closeness Centrality	.310 (.05)	.06
Number of triangles	13.21 (12.18)	.07*

*Note*.

*p < .05,

**p < .01,

***p < .001.

The EXITS multiple group memberships measure was not assessed in relation to only school group memberships. Mean scores are based on the whole sample (pre mean-centering) (*N* = 813).

To account for the hierarchical nature of school data, with students nested within year levels, we conducted linear mixed-effect model analyses in accordance with recommendations by Lee and Bryk [75]. This analytical technique allowed us to examine more closely the relative strength of multiple important group memberships (i.e., the index multiplying number of groups and importance of these groups) and each of the five network indices as predictors of PSE. Using the lmer package in R [76], we specified separate models to avoid collinearity between network measures and tested each of the network measures against the number of important groups as fixed explanatory variables. In keeping with recommendations by Barr and colleagues [77], in each equation we included terms for the random effects of year level on both the intercept and the slopes of all predictors. Standardized betas and bootstrapped confidence intervals are presented in Table 5. Inspection of the predictors showed that for each comparison, multiple important group memberships predicted PSE, but that the network measures made no additional contribution to prediction once group memberships were accounted for.

**Table 5:** Study 2: Standardized betas and confidence intervals of models comparing Multiple Important Group Memberships (MIGM) to individual network measures in predicting personal self-esteem, for children/adolescents in a boys’ school in Australia.

Network Measure	MIGM (β)	MIGM (CI)	Network (β)	Network (CI)
Out-Degree	.15*	.07 - .23	.07	-.03 -.17
In-Degree	.16*	.08 -.23	-.01	-.10 -.06
Degree	.16*	.07 - .24	. 03	-.05 - .10
Closeness Centrality	.16*	.08 - .25	-.01	-.11 - .06
Number of triangles	.16*	.08 - .25	.01	-.13 - .15

*Note*. MIGM = Multiple Important Group Memberships. Because of missing data on the PSE and the groups measures, *N* = 578 for these analyses.

* 95% CI does not include 0.

In line with H2, these results provide further evidence that it is the number of important groups and not the number of interpersonal ties that predict personal self-esteem. This is consistent with Study 1c's findings in so far as social connectedness was more strongly related to personal self-esteem when social connectedness was the basis for self-definition and identification with members of a meaningful social group or category [21,24].

## Study 3a: University Students in China

Study 1c provided evidence that after having been out of a homeless shelter for three months, it was multiple group memberships that best predicted personal self-esteem one year later. However, in that study, we were not able to examine directionality. Even though both paths are plausible—multiple group memberships might lead to higher personal self-esteem, or high personal self-esteem might lead to more group memberships [78]—theoretically, we are most interested in the former pathway.

To disentangle these two pathways, we therefore conducted a longitudinal study to assess whether it is multiple group memberships that predicts positive changes in personal self-esteem over time (and not the other way around, such that that personal self-esteem predicts positive changes in multiple group memberships over time). We tested this hypothesis among students at a large Chinese university and, given the cultural context, we operationalized self-esteem as self-competence [60].

### Method

#### Participants

The first wave was completed by 227 first-year undergraduates at a large university in China. Of those, 154 (40 male and 114 females with an average age of *M *= 19.76 years, *SD* = 1.02) took part in the second wave one month later. Participants had, on average, been at the university for 1.41 years (*SD* = .94). Results of the MCAR test confirmed that, on key measures, attrition was not systematic, but random, χ(3) = 4.613, *p* = .202.

#### Ethics Statement

This study obtained clearance from the ethics committee at the University of Wellington, New Zealand. In accordance with national guidelines, and with approval of the university ethics committee, participants read a statement before commencing the study that completion of the study implied consent. Reassurances were given about the anonymity of responses and the right to withdraw without penalty.

#### Procedure and Measures

Multiple group memberships were assessed by asking participants to indicate their agreement with two statements that were adapted from the previous studies (i.e., “At the moment, I am a member of lots of different groups” and “At the moment, I am active in lots of different groups”). To address positivity bias, two new items were included that were worded negatively: “I am currently a member of only one group” and “At the moment, I have strong ties with only one group” (1 = Do not agree, 7 = Agree completely). After reverse scoring the last two items, the four items were averaged (T1: α = .81; T2: α = .83).

Personal self-esteem was assessed using a self-competence measure. For this purpose we used the same three Personal Identity Strength items as in Study 1c (T1: α = .84 and T2: α = .85).

Various indicators of socio-economic status were again assessed. Participants were asked to record their family’s class background (1 = *Lower class*, 7 = *Upper class*), whether they were the first person in their family to go to university (1 = *Yes*, 2 = *No*), and the highest level of education that their parents achieved (on two scales ranging from 1 = *Grade school*, 7 = *Graduate degree*).

### Results and Discussion

Preliminary analyses revealed that the various socio-economic status indicators did not correlate with initial multiple group memberships or personal self-esteem (all *p*s > .225). We therefore did not control for these socio-economic indicators in our main analyses in this and subsequent studies that were conducted with university students.

Bivariate correlations are presented in Table 6. To examine the two pathways, we performed a regression analysis controlling for initial personal self-esteem at T1 when examining the relationship between multiple group memberships (T1) and personal self-esteem (T2). In line with H1, we found that multiple group membership at T1 significantly predicted personal self-esteem at T2, *β* = .15, *p* = .01. At the same time we found no support for the reverse relationship: personal self-esteem at T1 did not predict multiple group membership at T2 when controlling for multiple group membership at T1, *β* = .07, *p* = .288.

**Table 6:** Study 3a: Descriptive Statistics and Bivariate Correlations for Students in China.

	*M (SD)*	1	2	3	4
1. Multiple group memberships (T1)	4.68 (1.27)	1			
2. Personal self-esteem (T1)	4.86 (1.17)	.15	1		
3. Multiple group memberships (T2)	4.82 (1.24)	.63***	.15**	1	
4. Personal self-esteem (T2)	4.98 (1.11)	.25**	.73***	.22**	1

*Note*.

**p < .01,

***p < .001.

Correlations were based on a sample of *N* = 154.

There are two possible reasons why socio-economic status might not correlate with multiple group memberships and personal self-esteem as in previous studies [31,63]. First, this relationship may not generalize to the Chinese context under investigation. Second, the Chinese participants in this study had been at university for a longer time than those who took part in the studies by Iyer at al. [31] and Jetten et al. [70], possibly eroding the capacity of background socio-economic status to predict these outcomes.

In sum, this study provides longitudinal evidence that belonging to more groups at T1 is associated with higher personal self-esteem a month later (even when controlling for initial group memberships). We did not obtain any evidence for the reverse relationship such that initial levels of personal self-esteem are associated with more group memberships one month later. Thus, even though both relationships are theoretically plausible, this study only found evidence in support of H1.

## Study 3b: University Students in Australia

Study 3a provided evidence that multiple group memberships could account for change in self-esteem over time. However, in that study, we were not able to assess whether *change* in multiple group memberships over time was associated with subsequent change in self-esteem. To examine this possibility, we therefore conducted a longitudinal study across three time points to determine the extent to which change in multiple group memberships across the first semester of the study year could predict change in personal self-esteem over the full year (and not the other way around). We tested this hypothesis among students at an Australian university.

### Method

#### Participants

The first wave was completed by fourth-year students at the beginning of first semester (91 participants), the end of first semester (48 participants) and the end of second semester following their research thesis submission (94 participants). Of those, 47 (39 female, 8 male) with an average age of *M *= 23.41 years, *SD* = 5.71) took part in all three waves across 9 months.

#### Ethics Statement

The study obtained ethical clearance from the Behavioural and Social Sciences Ethical Review Committee (BSSERC) at the University of Queensland. Participants gave their written consent before they started completing the surveys.

#### Procedure and Measures

Multiple group memberships were assessed using the same measures as Studies 1a, 1b and 2 (T1: α = .93; T2: α = .94). Personal self-esteem was assessed using the one-item self-esteem scale used in Study 2.

### Results and Discussion

To examine the two pathways, we performed a regression analysis controlling for personal self-esteem at T1 as well as multiple group memberships at T1, when examining the relationship between multiple group memberships (T2) and personal self-esteem (T3). This allowed us to assess whether change in multiple group memberships predicted change in self-esteem. In line with H1, we found that multiple group memberships at T2 significantly predicted personal self-esteem at T3, *β* = .56, *p* = .005. We found no support for the reverse relationship: personal self-esteem at T2 did not predict multiple group memberships at T3 when controlling for multiple group membership and self-esteem at T1, *β* = .29, *p* = .127.

Similar to the analytical strategy used in Study 1c, we again calculated a gain score by subtracting T1 from T2 multiple group membership and correlated this with T3 personal self-esteem. Analysis revealed that gains in multiple group membership was positively related to T3 self-esteem (*r* = .297, *p* = .050).

In sum, by examining change over time in multiple group memberships and personal self-esteem, Study 3b provides greater evidence for the directional claim of H1: that it is changes in membership in important groups over time that predict personal self-esteem and not changes in personal self-esteem over time that predict membership in important groups.

#### Meta-analytical Integration

A meta-analysis was conducted on the data sets from Study 1a to Study 3b examining the overall cross-sectional relationship between multiple group membership and personal self-esteem. From the reported 6 studies, 10 effect sizes were calculated with a total sample size of 1,536 participants and correlations varying between .15 and .61. A random effect model with combined effect sizes while taking into consideration dependency between observations (i.e., correcting for dependent observations, accounting for the fact that some effect sizes are based on the same samples) revealed an overall weighted effect size of *r* = .309, 95% CIs [.223, .90], *Z* = 6.752, *p* <.001, representing a medium-sized effect [79]. In showing that multiple important group memberships are positively related to personal self-esteem, this finding consolidates support for H1.

## Study 4: University Students in the US

Studies 4 and 5 aimed to test mediation predictions—with a view to exploring the mechanism through which multiple important group memberships enhance personal self-esteem. More specifically, these studies tested H3: that the relationship between belonging to more important groups and higher personal self-esteem would be mediated by higher collective self-esteem.

Studies 4 and 5 differed in important ways from the previous studies. In particular, to increase control over the content of the group memberships, we provided participants with a list of group memberships and asked how important these group memberships were to them. In Study 4, participants were asked about three group memberships and, expanding the number of groups, in Study 5 participants were asked about seven group memberships. In both studies we assessed general social categories to which people belonged (e.g., nationality, gender) rather than smaller friendship groups to ensure that everyone would be part of the groups referred to.

### Method

#### Participants

Participants were 302 undergraduates (Age: *M* = 19.04, *SD* = 2.19; Gender: Male: *n* = 143, Female: *n* = 157, No response: *n* = 2) from the University of Kansas in the United States.

#### Ethics Statement

This study obtained ethical clearance from the ethics committee at the University of Kansas. Participants gave their written consent before they started completing the surveys.

#### Procedure and Measures

Participants completed the **questionnaire** at the beginning of the school year. They provided demographic information (i.e., age, ethnicity, year in school) before rating their identification with three different groups that they all belonged to—their gender, university sports team fan, and nationality. Specifically, identification with each group was measured in the way that best indexed that aspect of identification. Gender identification was measured with four items on an 8-point scale (e.g., “I like being a member of my gender group”, adapted from Schmitt and colleagues [80]; α = .89, *M* = 7.18, *SD* = 1.01), while identification with the university sports team was measured with seven items on an 8-point scale (e.g., “how strongly do you see yourself a fan of the [name of the university sports team]”, adapted from Wann & Branscombe [81]; α = .92, *M* = 5.38, *SD* = 1.70), and twelve items assessed identification with the United States on a 5-point scale (e.g., “I love my country”, α = .80, *M* = 3.87, *SD* = .55). An index of multiple important group memberships was computed by determining how many of these three identities participants rated as being of more than median-level importance. In this way, participants could rate none (*n* = 55), one (*n* = 101), two (*n* = 103), or all three (*n* = 43) of these group memberships as highly important to them.

Participants also completed the Rosenberg [30] *personal self-esteem* scale to indicate how they felt about themselves (10 items; e.g., I feel I have a number of good qualities; α = .88, *M* = 5.51, *SD* = 1.04). Following the reasoning of Branscombe and colleagues [26], we measured collective self-esteem using only two subscales from Luhtanen and Crocker's [44] *Collective Self-Esteem* (CSE) scale. Specifically, we combined four items from the Membership subscale (e.g., "I am a worthy member of the social groups I belong to") and four items from the Private Esteem subscale (e.g., "In general I'm glad to be a member of the social groups I belong to"; *M* = 5.34, *SD* = .80; overall α = .82). We did not include the Public subscale from the CSE measure because participants' views of how others perceive their group memberships were not relevant to our hypotheses. We did not use the Identity subscale from the CSE because this would overlap substantially with the identity measure that was used to determine multiple important group memberships. Both self-esteem measures were rated on 1 = *Strongly Disagree* to 7 = *Strongly Agree* scales, with higher scores indicating higher levels of esteem.

### Results

#### Collective Self-esteem

Regression analysis revealed that multiple group memberships positively predicted collective self-esteem, *β* = .30, *p* < .001; participants who were highly identified with a greater number of groups reported stronger collective self-esteem. A follow-up one-way ANOVA confirmed this linear relationship, *F* (1, 298) = 29.00, *p* < .001 (see Table 7). Participants who identified strongly with more groups reported higher collective self-esteem.

**Table 7:** Means and Standard Deviations as a function of the number of groups that participants highly identified with (*N* = 302, University students in the US, Study 4).

*Membership in important groups*	*Collective self-esteem*		*Personal self-esteem*	
	*M*	*(SD)*	*M*	*(SD)*
Zero	5.25	(.90)	5.03	(1.07)
One	5.59	(.87)	5.43	(1.12)
Two	5.79	(.77)	5.71	(.87)
Three	6.11	(.76)	5.80	(.99)

*Note*. Numbers in parentheses are standard deviations.

#### Personal Self-esteem

Consistent with H1, regression analysis revealed that there was a significant positive relationship between multiple group memberships and personal self-esteem, *β* = .25, *p* < .001, such that participants who were highly identified with a greater number of groups reported higher personal self-esteem. A follow-up one-way ANOVA revealed a significant linear effect for the number of groups that participants were highly identified with on personal self-esteem, *F* (1, 298) = 19.08, *p* < .001 (see Table 7).

#### Mediation Analyses

**Fig 2:**
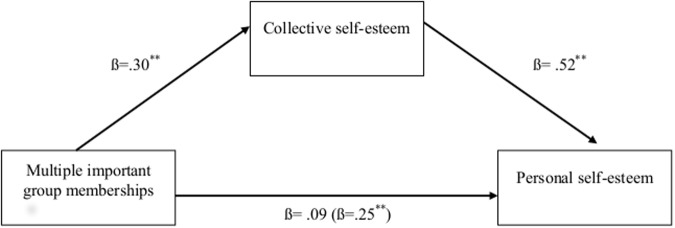
Study 4: The indirect effect of collective self-esteem on the relationship between multiple group memberships and personal self-esteem for University students in the US. *Note*. **p < .001. Correlations were based on a sample of *N* = 302. Beta within parentheses represents the direct effect.

To test H3 we sought to determine whether collective self-esteem could account for the relationship between multiple groups and personal self-esteem, by conducting a bootstrap analysis using 10,000 resamples. Supporting this hypothesis, collective self-esteem significantly mediated the effect of multiple important group memberships on personal self-esteem (IE = .17, *SE* = .039, 95% CI[.113, .238], see Fig 2). Bootstrapping assessing the reverse model revealed that personal self-esteem was also a significant mediator of the effect of multiple important group memberships on collective self-esteem (IE = .11, *SE* = .029, 95% CI[.068, .164]). However, theoretically, this model is less plausible than a model whereby CSE is the mediator of the relationship between important group memberships and personal self-esteem (i.e. it is theoretically unclear how personal self-esteem could affect collective self-esteem). Even though we do not consider this model further here, we suggest that future research should examine this process more fully by manipulating the proposed mediator experimentally.

### Discussion

Study 4 provides additional support for H1. Specifically, identifying with more groups was associated with higher levels of personal self-esteem. Moreover, in line with H3, we also found that collective self-esteem fully mediated the relationship between multiple group memberships and personal self-esteem. This is consistent with our argument that multiple group memberships lead individuals to feel good about themselves as individuals because they provide psychological resources that allow them to feel good about themselves as group members. Importantly, this effect was found in a context where participants were asked about their identification with broad social categories such as their gender, their university’s sports team, and their nation—arguably all group memberships that may not provide much tangible social support on a daily basis, but can be important in defining and locating the self in the world. In that sense, these groups are ideally suited to provide ‘grounding’ for the self.

## Study 5: University Students in the US

The aim of our final study was to again to test H3—that collective self-esteem mediates the relationship between the multiple groups an individual highly identifies with and personal self-esteem. We sought to replicate and extend the findings of Study 4 by focusing on a greater and more diverse range of groups.

### Method

#### Participants

Participants were 148 undergraduates (Age: *M* = 19.07, *SD* = 2.49; Gender: Male: *n* = 67; Female: *n* = 80; missing: *n* = 1) from the University of Kansas in the United States.

#### Ethics Statement

This study obtained ethical clearance from the ethics committee at the University of Kansas. Participants gave their written consent before they started completing the surveys.

#### Procedure and Measures

Participants completed a questionnaire at the beginning of the academic year in which they provided demographic information (i.e., age, ethnicity, year in university) before rating the importance to their self-concept of seven different group memberships (1 = *Not important to my identity*, 8 = *Very important to my identity*). These group memberships comprised ethnic (*M* = 4.20, *SD* = 2.24), student (*M* = 5.98, *SD* = 1.78), family (*M* = 6.67, *SD* = 1.68), sports fan (*M* = 4.20, *SD* = 2.34), gender (*M* = 6.45, *SD* = 1.77), national (*M* = 6.39, *SD* = 1.79), and religious (*M* = 4.95, *SD* = 2.47) groups. A measure of multiple group memberships was computed by assessing the extent to which participants rated the importance of these seven identities as higher than the median. In this way, participants could identify highly with either none (*n* = 10), one (*n* = 30), two (*n* = 24), three (*n* = 30), four (*n* = 16), five (*n* = 21), six (*n* = 11), or all seven (*n* = 6) of these groups.

As in Study 4, participants completed the 10-item Rosenberg [30] *personal self-esteem scale* (α = .88, *M* = 5.55, *SD* = 1.04). We again only included the Private Esteem and Membership subscales from Luhtanen and Crocker's [44] Collective Self-Esteem (CSE) scale (α = .87, *M* = 5.68, *SD* = .87). Both self-esteem scales were rated on a 7-point scale (1 = *Strongly Disagree*, 7 = *Strongly Agree*), with higher scores indicating higher levels of esteem.

### Results

Regression analysis revealed a significant positive relationship between multiple group memberships and collective self-esteem, *β* = .25, *p* = .002, such that participants who were highly identified with a greater number of groups reported higher collective self-esteem. A follow-up one-way ANOVA confirmed this significant linear effect, *F* (1, 140) = 9.85, *p* = .002. Consistent with H1, regression analysis also revealed a significant positive relationship between multiple group memberships and personal self-esteem, *β* = .20, *p* = .016, and this relationship was again confirmed in a follow-up one-way ANOVA testing the linear term, *F* (1, 140) = 5.97, *p* = .016. As in all our previous studies, participants who were highly identified with a greater number of groups reported feeling more positive about themselves.

**Fig 3:**
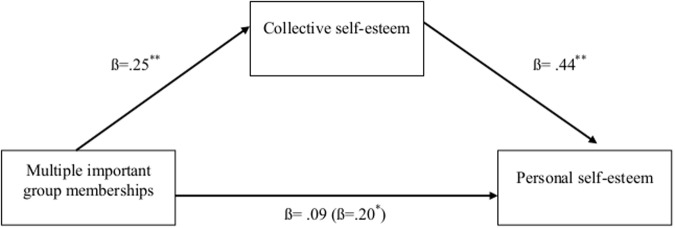
Study 5: the indirect effect of collective self-esteem on the relationship between multiple group memberships and personal self-esteem for University students in the US. *Note*. *p < .05, **p < .01. Correlations were based on a sample of *N* = 148. Beta within parentheses represents the direct effect.

As in Study 4, because high identification with an increasing number of multiple groups was associated with higher levels of both personal and collective self-esteem, a mediation model tested H3 to determine whether collective self-esteem can account for the relationship between multiple groups and personal self-esteem. In line with this hypothesis, bootstrap analysis using 10,000 resamples revealed that collective self-esteem significantly mediated the effect of multiple important group memberships on personal self-esteem (IE = .06, *SE* = .027, 95% CI[.025, .114]) (see Fig 3). We also tested the reverse model: whether personal self-esteem mediated the relationship between multiple group memberships and collective self-esteem. Bootstrapping testing the reverse model revealed that personal self-esteem was also a significant mediator of the effect of multiple important group memberships on collective self-esteem (IE = .04, *SE* = .021, 95% CI[.011, .081]). Again, because this model is theoretically less plausible than the model treating CSE as mediator, and because the alternative model is weaker than the predicted model, we do not discuss this model further.

### Discussion

Study 5 replicates the findings of Study 4, this time using a greater and more varied range of social categories. As in all our previous studies, we found support for H1 such that identifying with multiple groups was associated with higher levels of personal self-esteem. In line with H3, we also found that collective self-esteem can account for the relationship between multiple groups and personal self-esteem—supporting the idea that one reason why multiple group memberships make people feel good about themselves as individuals is that those groups provide resources that make them feel good about themselves as group members.

## General Discussion

In this paper we tested the proposition that group memberships are an important social resource that enhances self-esteem. Specifically, we examined the hypothesis that identification with multiple important social groups provides a basis from which to draw psychological resources to boost personal self-esteem. This prediction (H1) was examined in eight studies, and all provided clear evidence of the 'more the merrier’ effect; such that the more groups a person identified with, the higher their self-esteem. Our meta-analysis across the first 6 studies revealed a medium-sized effect of .307. Moreover, this relationship was found to hold across a range of populations ranging from British children (Study 1a), older adults in China (Study 1b), former residents of a homeless shelter (Study 1c), schoolboys in Australia (Study 2), to Chinese, Australian and American university students (Studies 3a, 3b, 4 and 5).

In addition, we demonstrated that the effects of multiple important group memberships on personal self-esteem are not reducible to the number of one’s interpersonal ties. In line with H2, Study 1c thus found that when comparing the predictive power of multiple important group memberships with that of the number of individuals that residents of a homeless shelter listed as important in their life, only the former significantly predicted personal self-esteem. Study 2, confirmed this finding in a comprehensive social network analysis of Australian schoolboys that compared the ability of multiple important group memberships and the number of strong interpersonal ties to predict personal self-esteem. Again, we found that in analyses where the relative strength of these measures were compared it was the number of important group memberships, and not the number of important interpersonal ties, that was the better predictor. This finding is consistent with our theorizing that multiple important group memberships boost personal self-esteem more than important interpersonal ties because the former allow for a self-definition as ‘we’, linking the individual self to the group, which permits the individual to benefit from unique group resources [21,56].

We also provide evidence for directionality. In Study 1c, preliminary support for the role of multiple important group memberships in predicting personal self-esteem was found seven months later; but not the other way around. Additional support for the direct influence of multiple group membership on self-esteem one month later was obtained in Study 3a. Again, there was no evidence for the reverse relationship in which personal self-esteem predicts multiple group membership over time. Yet it was the findings of Study 3b that provided the strongest evidence for directionality. Here, over three measurement points, we found that it was changes in multiple group memberships over time that predicted changes in personal self-esteem. We found no evidence for the reverse model whereby changes in personal self-esteem predict changes in multiple group memberships over time.

Going further still, Studies 4 and 5 provided support for a mediation hypothesis (H3), in which membership in multiple important groups boosts personal self-esteem because those memberships provide resources (a sense of belonging, meaning and purpose) that form the basis for collective self-esteem. In this way, we suggest that one key pathway through which people come to feel good about themselves as individuals is by feeling good about themselves as group members—something that is more pronounced the more groups they identify with.

### Theoretical Implications

All eight studies provide evidence for what is arguably an assumption that lies at the heart of social identity theorizing: important group memberships provide psychological resources. Therefore, when individuals have these resources, it should improve how they feel about themselves. This insight aids our understanding of how mere identification with social groups boosts personal self-esteem. There are two implications in particular that we would like to focus on.

First, our findings can help to explain when and why identities are associated with positive well-being outcomes and when no such relationship can be expected. While the notion that identities provide resources is well-established [11,82] empirical evidence of the relationship between multiple identities and well-being is mixed. Specifically, while there is a vast body of work showing that multiple identities enhance well-being [31], there is also considerable work, including a meta-analysis of the self-complexity literature [83] suggesting that having more identities is associated with lower well-being. Reviewing the self-complexity literature, it becomes clear that identity is defined rather loosely in this research, comprising not only social identities derived from group membership, but also social roles, and traits that are perceived as self-defining (e.g., honesty).

By shifting the focus to the extent to which individuals perceive that they are *psychologically* connected or linked to a group [21,24,57], we bring the question back to a quite basic consideration: “is this relationship important for defining who I am?” It is only when people have internalized a relationship, role, group membership or social category as an important part of self that it will have the capacity to predict self-esteem. This is because others are now *part of the self*, encompassed in an individual’s social identity [15].

Second, our findings shed light on the way in which group memberships and social identities affect outcomes at a personal level. In this regard, our findings show that self-esteem is not solely a ‘personal’ aspect of self. Instead, it is not only part of, but also determined by our social identities [84]. Once we recognize that self-esteem is partly conditional on group memberships that determine our sense of identity, this opens up a whole new way of thinking about self-esteem—in particular, by pointing to the interconnectedness of different levels of self-understanding. Consistent with classic views that the self is primarily social [85] this has the potential to help develop richer theoretical models of how the social context and our engagement with social groups affect outcomes that are often seen as “deeply personal” [86].

### Limitations and Future Research

The current work has a number of limitations that also provide for promising future research directions. One potential limitation of our research is that we did not provide direct evidence for our psychological resource model. That is, even though membership in additional important groups counts and contributes to personal self-esteem, we did not provide direct evidence that the construct we have termed *psychological resources* mediates the relationship between multiple important group memberships and personal self-esteem. Even though the empirical case would have been strengthened by showing such mediation, the challenge lies in specifying the actual resource(s) that group membership provides. Such evidence would also help to counter alternative explanations for our findings. For instance, one can argue that the importance of each group membership diminishes with each added group membership. This then reduces the power of groups over the individual, thus enhancing his or her sense of personal freedom and ultimately self-esteem.

Thus, while we argue that group membership furnishes people with a sense of belonging, purpose, meaning, and grounding, it is not entirely clear whether these are the only and most important resources associated with multiple group memberships. In addition, it is not clear what measure would tap all of these psychological resources. Arguably too, even if it were possible to delineate this construct, it is questionable whether any measure would be sufficiently sensitive to tap it given that many participants may not be consciously aware of the resources associated with group membership.

It is also important to consider other ways that future research might examine the psychological resource model. For example, researchers may consider a moderation model and manipulate the extent to which group membership provides collective self-esteem. According to the psychological resource model, membership in important groups should only enhance personal self-esteem to the extent that collective self-esteem can be derived from group membership.

Another potential limitation of our research is that we did not provide experimental evidence for our main prediction that membership in important groups boosts personal self-esteem. Such evidence is essential to isolate the effects of group membership from other effects that may influence the relationship between membership in important groups and self-esteem (e.g., social support). However, even though we agree that it may be important to show evidence for causality by providing experimental evidence, obtaining such evidence is unlikely to be straightforward. Even though Jones and Jetten [38] showed that the mere salience of a greater number of group memberships is associated with resilience, such experimental effects have so far only been found on behavioral measures (e.g., withstanding pain in a cold pressor task) and there is no research showing these effects on self-report measures. We suspect that because the effect of group membership on self-esteem is likely to be largely unconscious, it may be difficult to tap this process using explicit self-esteem measures. Furthermore, even though there is evidence that the importance of group memberships can be manipulated using false feedback procedures [87,88], it is also clear that the effects of such manipulations are relatively weak. These manipulations may therefore not be sufficiently powerful to vary the extent to which group memberships are perceived as psychological resources. Finally, even though personal self-esteem can be operationalized as both a trait and a state construct [18], there may be limits in the extent to which salience manipulations of multiple important group memberships are powerful enough to affect levels of trait personal self-esteem. In sum, even though all three considerations lead us to suspect that it may be difficult to provide experimental evidence for H1, given the need for studies providing evidence for causality, it might be worthwhile attempting to do so while keeping these considerations in mind.

Finally, we acknowledge that not all important group memberships have the capacity to furnish members with a positive identity via enhanced collective self-esteem. Consistent with this notion, a large literature has shown that particular devalued or stigmatized identities can be a liability, as evident in the negative health and well-being outcomes when a person either identifies with these groups [89] or reveals their membership in these groups [90]. Despite this, most research has shown the opposite effect: that identification with a minority group serves not only as a buffer against environmental threats (e.g., discrimination, exclusion, poverty), but is a key resource that can be mobilized in managing and combating exclusion and discrimination. Such increased identification in response to perceived discrimination has clear psychological benefits that at least partially counteract the negative effects of perceived discrimination on well-being [26,91,84]. There is also evidence that group members flexibly use their group memberships, to the extent that when one identity is threatened, they can easily fall back on another [92]. Future research should examine the way that group members utilize the psychological resources that are associated with the groups they belong to, and test whether any negative personal self-esteem consequences associated with one identity can be offset by identification with another group. This is particularly important because it is individuals who face stressors such as discrimination and stigma who are likely to benefit most from having access to identity resources that allow them to cope effectively.

### Concluding Comment

Our work speaks to a basic assumption in social identity theorizing—that group membership has the potential to provide members with a positive identity and that this has beneficial effects for self-esteem [14]. Even though this is an established insight, the novel contribution of our work is that it provides the first empirical test of the classic social identity theory premise by showing that the individual and their own perceived self-worth is affected by membership in social groups in profound ways. In this way, we hope that our research will serve to rekindle group researchers’ interest in self-esteem and give impetus and direction to an important new seam of psychological enquiry.
