# Correction: Having a Lot of a Good Thing: Multiple Important Group Memberships as a Source of Self-Esteem

**DOI:** 10.1371/journal.pone.0131035

**Published:** 2015-06-15

**Authors:** Jolanda Jetten, Nyla R. Branscombe, S. Alexander Haslam, Catherine Haslam, Tegan Cruwys, Janelle M. Jones, Lijuan Cui, Genevieve Dingle, James Liu, Sean C. Murphy, Anh Thai, Zoe Walter, Airong Zhang

The tenth author’s name is misspelled. The correct name is: Sean C. Murphy.
